# Type I Interferon (IFN)-Regulated Activation of Canonical and Non-Canonical Signaling Pathways

**DOI:** 10.3389/fimmu.2020.606456

**Published:** 2020-11-23

**Authors:** Candice Mazewski, Ricardo E. Perez, Eleanor N. Fish, Leonidas C. Platanias

**Affiliations:** ^1^ Robert H. Lurie Comprehensive Cancer Center of Northwestern University, Chicago, IL, United States; ^2^ Division of Hematology-Oncology, Feinberg School of Medicine, Northwestern University, Chicago, IL, United States; ^3^ Toronto General Hospital Research Institute, University Health Network and Department of Immunology, University of Toronto, Toronto, ON, Canada; ^4^ Department of Medicine, Jesse Brown Veterans Affairs Medical Center, Chicago, IL, United States

**Keywords:** interferon, signaling, MAP kinase signaling, signal transducer and activator of transcription, mammalian target of rapamycin, mRNA translation, SARS-CoV-2, COVID-19

## Abstract

For several decades there has been accumulating evidence implicating type I interferons (IFNs) as key elements of the immune response. Therapeutic approaches incorporating different recombinant type I IFN proteins have been successfully employed to treat a diverse group of diseases with significant and positive outcomes. The biological activities of type I IFNs are consequences of signaling events occurring in the cytoplasm and nucleus of cells. Biochemical events involving JAK/STAT proteins that control transcriptional activation of IFN-stimulated genes (ISGs) were the first to be identified and are referred to as “canonical” signaling. Subsequent identification of JAK/STAT-independent signaling pathways, critical for ISG transcription and/or mRNA translation, are denoted as “non-canonical” or “non-classical” pathways. In this review, we summarize these signaling cascades and discuss recent developments in the field, specifically as they relate to the biological and clinical implications of engagement of both canonical and non-canonical pathways.

## Introduction

Established cellular signaling pathways have been referred to in the context of canonical or “classical” and non-canonical or “non-classical” signaling cascades that control distinct outcomes in the cell. A canonical pathway indicates the conventional protein signaling, typically considered the main effect or, maybe more appropriately, the first effect discovered and elucidated; non-canonical pathways are alternative pathways to the canonical, but that should not imply less importance (1). Perhaps, the most well-described signaling in terms of canonical and non-canonical pathways is Wnt signaling, specifically the canonical β-catenin pathway (2). Additionally, inflammation and immunoregulatory related pathways such as nuclear factor-κB (NF-κB) and interferon (IFN) signaling are described as canonical and non-canonical (3, 4). Recent discoveries of additional non-canonical pathways, some that interconnect with canonical signaling, add to the complexity surrounding different biological outcomes.

The IFNs are cytokines that can be divided into three groups: type I (IFNα, IFNβ, IFNδ, IFNϵ, IFNκ, IFNτ, IFNω, and IFNζ), type II (IFNγ), and type III (IFNλ) (5). Type I IFNs were first discovered in 1957, followed by type II in 1965, while much more recently, in 2003, type III IFNs were identified (6–8). Type I IFNs have the most family members. The predominant type I IFN subtypes studied are IFNα and IFNβ, partially due to IFNδ, IFNτ, and IFNζ not having human homologs, more specific cellular sources of IFNϵ and IFNκ, mainly female reproductive organs and keratinocytes, respectively, and IFNω being studied more in felines (5, 9, 10). The roles of IFNα and IFNβ in antiviral responses have been most reported, but these type I IFNs also have significant relevance in cancer and autoimmune diseases (11–13).

Production of type I IFNs is induced by pathogen-associated molecular patterns, viral RNA or DNA fragments, and is associated with activation of pattern recognition receptors (11). Once activated, the receptors initiate signal transduction that involves adapter proteins, eventually leading to activation and translocation of IFN regulatory factor 3 (IRF3) and NF-κB, which promote type I IFN production either directly or indirectly through IRF7 (11). IFNα is mainly produced by plasmacytoid dendritic cells (pDCs), whereas IFNβ is ubiquitously produced by immune cells (13).

Following transcriptional activation and mRNA translation, type I IFNs are secreted from immune cells and, on neighboring cells, bind to the two cellular receptor subunits IFNα receptor 1 (IFNAR1) and IFNAR2, which are associated with tyrosine kinases TYK2 and Janus kinase 1 (JAK1), respectively (9). Dimerization of the receptor initiates the autophosphorylation of JAK1, which phosphorylates and activates signal transducers and activators of transcription 1 (STAT1) and STAT2 proteins, which form a complex with IRF9, resulting in a well-characterized complex, IFN-stimulated gene factor 3 (ISGF3). ISGF3 translocates to the nucleus where it binds to IFN-stimulated response elements (ISREs) in the promoters of genes, leading to transcription of IFN stimulated genes (ISG) (14). Additionally, JAKs can phosphorylate and initiate the formation of phosphorylated STAT complexes of STAT1 and STAT3 homodimers, where the STAT1 homodimer is associated with a pro-inflammatory response, mediated by binding to gamma activated sequences (GAS), and the STAT3 homodimer indirectly inhibits inflammatory gene expression, restraining pro-inflammatory responses (15). These JAK/STAT IFN-signaling pathways are considered the canonical pathways. In addition, type I IFNs have also been reported to activate the formation of STAT2:STAT3 heterodimers and a STAT5:CrkL complex, invoking transcriptional activation of ISGs (16, 17).

**Figure 1:**
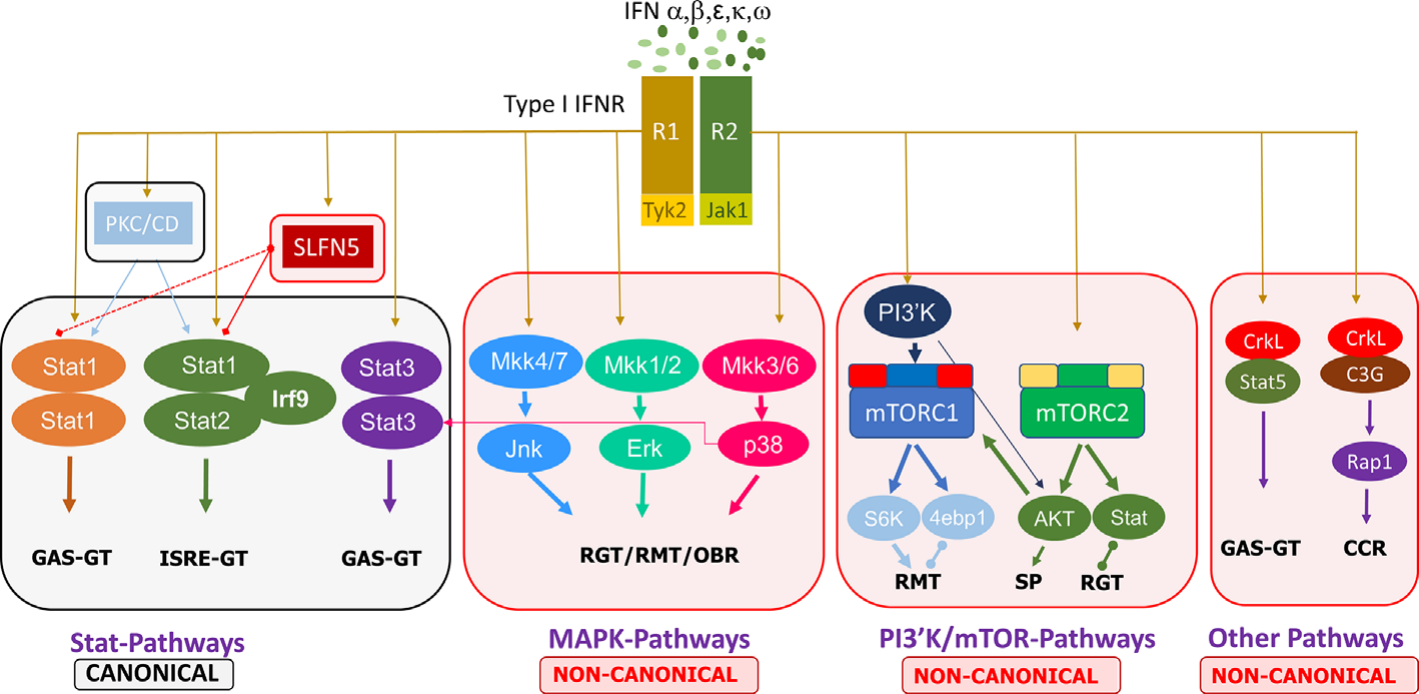
Summary of the canonical and non-canonical pathways involved in type I interferon signaling. 4ebp1, eukaryotic translation initiation factor 4E binding protein 1; CCR, cell cycle regulation; ERK, extracellular signal-regulated kinase; GAS, gamma-activated sequence; GT, gene transcription; IFN, interferon; IFNR, interferon receptor; IRF, interferon regulatory factor; ISRE, interferon-stimulated response element; JAK, janus kinase; Jnk, c-Jun N-terminal kinase; MKK, mitogen activated protein kinase kinase; mTORC, mammalian target of rapamycin complex; PI3′K, phosphoinositide 3-kinase; PKC, protein kinase C; CD, Calmodulin-dependent kinase, R1/R2, receptor 1/2; Rap, Ras-related protein; RGT, regulation of gene transcription; RMT, regulation of mRNA translation; OBR, other biological responses; S6K, ribosomal protein S6 kinase; SLFN, Schlafen; STAT, signal transducer and activator of transcription; SP, survival pathways.

Non-canonical type I IFN signaling pathways are similarly activated by IFNs binding to the extracellular regions of the dimeric IFNAR1 and IFNAR2 complex, leading to JAK1/TYK2 activation, but diverge from that point, specifically, not involving STAT activation by the JAKs. Evidence points to the regulation of STATs by non-canonical modifiers, with serine phosphorylation of STATs versus the tyrosine phosphorylation by JAK1/TYK2 (18). The main non-canonical IFN pathways identified thus far are the MAP kinase (MAPK) and phosphoinositide 3-kinases (PI3K)/mammalian target of rapamycin (mTOR) pathways, but there are other non-canonical modifiers such as SIRT2 and the Schlafen (SLFN) family (18, 19). MAPK and PI3K/mTOR pathways have been shown to elicit effects on ISG transcription and mRNA translation while also having some interaction with STATs in the canonical cascade (18). Further discoveries on the effectors of these pathways, such as the importance of Unc-51–like kinase (ULK1) in MAPK type I IFN-induced signaling, add to the complexity of type I IFN signaling cascades and demonstrate that the focus cannot be limited to the classical pathways (20). Other non-canonical modifiers include SLFN family members. Type I IFNs upregulate SLFN gene expression, and SLFN5 interaction with STAT1 has been demonstrated, indicating its effect downstream of JAK1 (21). SLFNs have been shown to be involved in antiviral responses, and their high expression in specific human immune cell subsets has been identified, such as elevated SLFN5 in T cells (22). These non-classical IFN-induced effectors have critical roles in ISG transcription, independent of or in conjunction with the canonical pathway, eliciting specific biological responses. A summary of the canonical and non-canonical pathways of type I interferon signaling is shown in **Figure 1**.

Below we provide an update on type I IFN canonical and non-canonical signaling, related to antiviral responses, antiproliferative effects in cancer, and immune regulation in autoimmune diseases, focusing on studies within the last few years. We address the type I IFN response to SARS-CoV-2 and the potential for therapeutic use for COVID-19.

## Biological Effects in Diseases

### Canonical and Non-canonical IFN Signaling in Malignancies

Type I IFNs have been studied in a wide range of cancers in the last few years, as illustrated in **Table 1**. These studies have focused exclusively on the IFNα and IFNβ subtypes, demonstrating their clinical relevance over other type I IFN subtypes. The signaling analyses in the last few years have still focused more on the JAK/STAT cascades, specifically STAT1 effects in type I IFN signaling. However, some reports explored the impact of STAT3 versus STAT1, as well as the non-canonical involvement of MAPKs, SIRT2, and SLFN5.

**Table 1:** Canonical and non-canonical type I interferon signaling in malignancies.

Type of cancer****	IFN pathway (canonical or ****non-canonical)	****Type I IFN (related) used/analyzed	****Methods/models	****Main results	Ref.****
Bladder	Non-canonical—MAP3K8 (TPL2)/ERK	IFNα	*In vitro* bladder - T24, 5637, HEK293A *In vivo:* T24 or 5637 cells SC into flanks BALB/c (nu/nu*):* mice – IFNα, roflumilast *Clinical:* MIBC tissue microarray chips (n=126) *Bioinformatics:* TGCA & Oncomine	-IFNα decreased COX-2, TPL2, ERK, IKK α/β, & cAMP levels but little effect on JAK/STAT -TPL2 co-IP with IFNAR2 (not IFNAR1), IFNα & TPL2i decreased pTPL2-IFNAR2 -IFNα + roflumilast synergistically suppressed tumor growth, cAMP & PGE2 sera levels in mice	(23)
Cervical	Canonical—JAK/STAT1, 2, & 3	IFNα2 IFNβ (IFNAR1/2)	*In vitro:* HeLa human cervical cancer cells, KO clones: IFNAR1, IFNAR2, STAT1, STAT2, STAT1 + STAT2 dKO, STAT2 + IRF1 dKO, & STAT3	-KO of IFNAR1 or 2 inhibited p-STATs & ISGs -STAT1 or 2 KOs had low ISG & dKO blocked ISG -STAT3 KO had no effect on ISG, p-STAT1 or 2, or IFNβ induced negative feedback regulators	(24)
Colorectal	Canonical or non-canonical—STAT3	IFNα IFNβ (IFNAR1)	*Bioinformatics:* TCGA dataset *In vitro:* Murine colon carcinoma -MC38 *In vivo:* IFNAR1-KO, IFNAR1-TKO, WT C57BL/6 & SJL mice - MCA or MC38 SC *Clinical:* Peripheral blood - healthy donors SCBC, CRC tissues - GCC	-Tumors grew faster & larger in IFNAR1-KO mice vs WT & in IFNAR1-TKO vs WT -Inhibition of p-STAT3 (not p-STAT1) decreased a granzyme B expression increase by IFNα/β in CTLs	(25)
Glioma	Non-canonical—SLFN5-STAT1	IFNα IFNβ	*Bioinformatics:* GlioVis Database *In vitro:* GBM - LN18, LN229, LN443, U87MG, MBM - DAOY & D556, PDX derived GSC	-SLFN5 expression increased at basal levels & further induced by IFNα or IFNβ in PDX glioma stem cell & established GBM & MBM cells -SLFN5 co-IP'd with STAT1, not STAT3 or 5, in 293T cells & signal increased with IFNβ treatment	(26)
Hepato-cellular	Non-canonical—SHP2/STAT1	IFNα	*In vitro*: hepatocellular - HepG2, Huh7, human embryonic kidney- HEK293A *In silico*: SHP2 & quercetin computational docking	-Quercetin increased IFNα induced p-STAT1 & ISG expression & decreased SHP2 expression in HepG2 -SHP2 overexpression decreased IFNα (+ quercetin) ISRE reporter expression in HepG2	(27)
Leukemia, lymphoma	Non-canonical—SIRT2/CDK9	IFNα IFNβ	*In vitro*: leukemia – HEL, KT-1, lymphoma - U937, Sirt2+/+, Sirt2−/−, Sirt1+/+, Sirt1−/−, Sirt6+/+, and Sirt6−/− MEF	-Sirt2−/− MEF had no IFNβ induced STAT1 activation or expression of ISG (Oasl2 Cxcl10 ISg15, ISg54) -SIRT2 regulated IFNβ induced CDK9-mediated p-STAT1 -SIRT2 KD leukemia cells less sensitive to IFNα–mediated antiproliferative effect	(19)
Leukemia, lymphoma, myeloma	Non-canonical—ULK1	IFNβ	*In vitro*: leukemia—KT-1, lymphoma—U937, myeloma—U266, Akt1/2+/+, Akt1/2−/−, Ulk1/2+/+ & Ulk1/2−/− MEFs	-IFNβ induced p-ULK1 Ser757 (mTORC1 phospho site) -ULK1/2 KO reduced ISRE & GAS activity & IFNβ induced ISG transcription, p38 activation, & antiproliferative effects	(20)
Melanoma	Canonical—JAK/STAT1	IFN-α2b	*Clinical*: NCT01460875 – SC IFN-α-2b 3/week 10 MU/M^2^—4 weeks, dose reduction every two weeks after first month—total 11 months	−91% of patients had stable or increased p-STAT1 levels over time of dose reduction -ISGs (OAS1 CXCL10, CD69 and SOCS1), not significantly less at end/ with lower IFN-α-2b dose -Higher p-STAT1 after initial dose had lower recurrence	(28)
Melanoma	Canonical—JAK/STAT1	IFNα IFNβ	*In vitro:* mouse melanoma - B16-OVA; Me-ALA incubation + irr *In vivo*: C57BL/6 and IFNAR1−/− mice; dendritic cells collected from bone marrow	-PDT of melanoma cells increased IFNα/β and apoptosis -PDT increased cGAS receptor (not MDA-5, TLR3, RIG-1), p-STAT1 & ISGs (CXCL10, ISG15, MX1) -WT DCs migrated toward PDT melanoma cells more than IFNAR−/− DCs	(29)
Ovarian	Canonical—JAK/STAT	(IFNAR1 ISG15),	*In vitro:* ID8-Defb29/Vegf-a mouse ovarian cancer cells - AZA *In vivo:* Pre-treated & ID8-VEGF-Defensin cells IP in C57BL/6 or NSG mice - AZA & anti-IFNAR1 IP	-anti-IFNAR1 inhibited AZA induced anti-tumorigenic response, survival benefit, increase in CD45+ immune cells, activation of CD8+ T and NK cells, & increase in ISG15 in immunocompetent mice but not in NSG mice	(30)
Immune focused	Non-canonical—p38/STAT3	IFNα	*Ex vivo* mDC isolated from PBMCs from human blood (MBDS*):*, DC/T cell co-culture *In vivo:* C57BL/6 mice—IFNα IP	-IFNα upregulates PD-L1 expression on myeloid immune cell & T-cell populations & on DC in mice -IFNα increased p38 and STAT3 activation & STAT3i & p38i (not PI3Ki or ERKi) decreased IFNα induced PD-L1 expression in mDC	(31)
Multiple	Canonical—JAK/STAT1	IFNα IFNβ	*In vitro*: breast—MCF-7, Hs578T, SK-BR-3, HCC70, T47D, melanoma - MDA-MB-435, Squamous - SCC61, Nu61, MES glioma cells *In vivo*: MCF-7 pre-treated anti-APO-1 or IFNβ injected into fat pad NGS mice	-Long term CD95 stimulation induced type I IFNs, p-STAT1, & increased ISGs in cancer cells -CD95L or type I IFN increased stemness and sphere formation in MCF-7 & SCC61, blocked by JAK1/JAK2i -p-STAT1 correlates with cancer stemness & KO of STAT1 blocked CD95L or type I IFN induced stemness	(32)
Multiple	Canonical—JAK/STAT1	IFNβ (IFNAR1)	*In vitro* melanoma—B16F10 lung—TC-1, lymphoma—YAC-1, thymoma—EG7 *In vivo:* C57BL/6, Ly5.1þ & IFNAR1–/– mice, LCMV-clone 13 (Cl13),: IP or IV, anti-CD4	-Chronic Cl13 infection lead to elevated IFNβ in sera -STAT1 mRNA higher in NK & protein expression higher in NK & T cells from Cl13-infected mice -Anti-IFNAR1 increased tumor metastasis 20% in Cl13-infected mice	(33)

STAT1 phosphorylation and the induced expression of various ISGs such as *OASL* and *ISG15* have commonly been used as indicators of a type I IFN response (19, 27, 28, 34, 35). In a study on cervical cancer, the importance of IFN-inducible activation of STAT1 and STAT2 was demonstrated through the use of STAT1 and STAT2 knockout human HeLa cells, yet the STAT3 knockout did not have any effect on ISGs (24). By contrast, in colorectal cancer, inhibition of p-STAT3 but not p-STAT1 decreased IFNα and IFNβ induced granzyme B expression in cytotoxic T lymphocytes (25). These differences highlight how different effectors activated by type I IFNs are dependent on cell type and disease specificity. Bazhin et al. also explored IFN-activated STAT3 effects, identifying a non-canonical interaction with p38 MAPK on STAT3 phosphorylation in mature DCs (31). ULK1 has been identified as a regulator of p38 MAPK and ISGs, downstream of mTOR, in type I IFN signaling in myeloproliferative neoplasms (20). This demonstrates a connection between both major IFN activated non-canonical signaling pathways. Another MAPK, extracellular signal-regulated kinase (ERK), is involved in non-canonical type I IFN signaling in malignancy, where mitogen-activated protein kinase kinase kinase 8 (MAP3K8) and ERK phosphorylation were decreased upon IFNα treatment in bladder cancer cells (23). Further elucidation is needed on the STAT-dependent and -independent non-canonical functions of the many MAPK pathway proteins.

Additional effects of non-canonical type I IFN-induced signaling in various malignancies have been examined. A glioblastoma study identified SLFN5 as a regulator of STAT1 induction by type I IFNs (26). In leukemia and lymphoma cells, type I IFN induced phosphorylation of STAT1 on serine 727 is mediated by cyclin dependent kinase 9 (CDK9), and this activation is dependent on the deacetylation of CDK9 by SIRT2 (19). Additionally, quercetin, a natural compound, decreases Src Homology Phosphatase 2 (SHP2), a negative regulator of STAT1 (27).

An important issue related to the clinical use of IFNs is toxicity and adverse events. Although approved in 1986 by the FDA for the treatment of malignancies and viral disorders, with demonstrated positive disease outcomes, IFNα is currently not commonly used in cancer treatment due to adverse effects (36, 37). A pilot study looked at the potential of decreasing the dose of IFN-α2b for the treatment of melanoma over the course of an 11-month treatment period. Despite the dose reduction, p-STAT1 levels were induced at comparable levels throughout the 11 months, and the IFN was well-tolerated (28). An alternative strategy has been to stimulate the endogenous type I IFN response in immune cells. Tsuchiya et al. genetically engineered induced pluripotent stem cell (iPSC)-derived proliferation myeloid cells (iPSC-pMCs) to produce IFNα. When injected into mice, these IFN-producing iPSC-pMCs exerted immunomodulatory effects analogous to direct type I IFN administration, yet without adverse effects or hematopoietic stem cell exhaustion (37). Brown et al. studied recombinant poliovirus/rhinovirus chimera PVSRIPO effects in cancer immunosuppression and found PVSRIPO infection of DCs increased IFNβ production and a sustained type I IFN response, as indicated by p-STAT1 and ISG induction (*IFIT1*, *ISG15*) (34). In a separate study, the use of photodynamic therapy (PDT) lead to the upregulation of type I IFNs in melanoma cells and DCs co-cultured with the PDT treated cells; the authors proposed this *ex vivo* strategy of stimulating DCs with the use of PDT as a possible immunotherapy (29).

Distinct from the positive outcomes of type I IFN treatment for malignancies, a number of studies have addressed the potential link of IFN treatment with chemotherapy resistance, immunosuppression, and driving of cancer stemness. Qadir et al. found chronic CD95 activation leading to cancer stemness was driven by IFNα/β-STAT1 canonical signaling (32). They also provided evidence that radio-resistant squamous cancer cells had increased p-STAT1 and ISG expression and that type I IFN treatment of breast and squamous cancer cells increased stemness and sphere formation, which was blocked by JAK inhibition, indicative of the involvement of canonical signaling.

Several studies have evaluated the effects of type I IFN administration in combination with immunotherapy. One group showed that IFNα increased programmed death-ligand 1 (PD-L1) expression on various immune cells through non-canonical p38/STAT3 signaling (31). The inference is that combining immunotherapy with IFNα treatment would limit the immunosuppressive effects of IFN treatment and permit effective growth inhibition. Similarly, another study provided evidence that IFNα-iPSC-pMC treatment increased PD-L1 mRNA, and when combined with a PD-L1 inhibitor, synergistic anti-tumor effects were reported (37). The poliovirus/rhinovirus type I IFN induced response likewise increases PD-L1 expression (34). Additionally, a bioinformatics examination of IFN gene deletions revealed that homozygous deletion of IFN was significantly associated with non-response to anti-CTLA4 treatment among melanoma patients (38). Overall, these studies suggest type I IFNs may have a critical role in immunotherapy strategies, possibly *via* a combination of type I IFN treatment with PD-L1 inhibition. Moreover, the data suggest that PD-L1 expression may be affected by IFN-induced non-canonical signaling.

### Canonical and Non-canonical Signaling in Autoimmune Diseases

**Figure 2:**
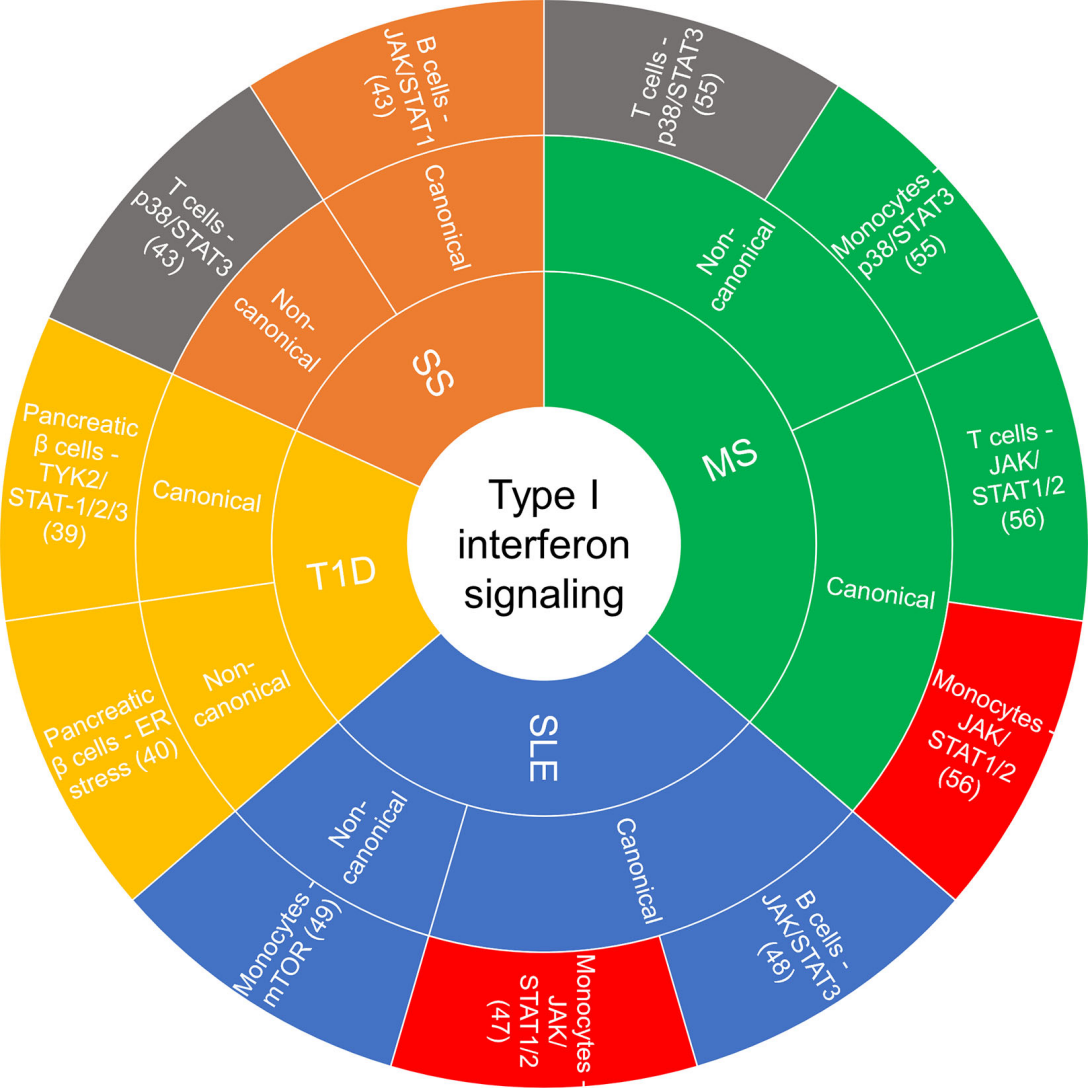
Sunburst chart of cell-specific canonical and non-canonical signaling recently reported on in autoimmune diseases. If there was a commonality in a canonical or non-canonical signaling demonstrated in the same cell type but different disease, they were color coded the same: red—JAK/STAT1/2 in monocytes, gray—p38/STAT3 in T cells. ER, endoplasmic reticulum; JAK, janus kinase; MS, multiple sclerosis; mTOR, mammalian target of rapamycin; SLE, systemic lupus erythematosus; SS, Sjögren’s syndrome; STAT, signal transducer and activator of transcription; T1D, type I diabetes.

Accumulating evidence implicates chronic and persistent type I IFN signaling in systemic inflammation that promotes the pathogenesis of some autoimmune diseases, including systemic lupus erythematosus (SLE), rheumatoid arthritis, multiple sclerosis (MS), type I diabetes (T1D) and Sjögren’s syndrome, among others (13, 39). These conditions are associated with different clinical symptoms and management strategies, yet there are common features related to the underlying inflammatory signaling pathways involved and the dysregulated immune response. **Figure 2** summarizes the cell type-specific type I IFN-induced canonical and non-canonical signaling pathways recently implicated in autoimmune diseases.

IFNα has been shown to impact the onset and progression of T1D, which involves the autoimmune attack of pancreatic β cells (40). One study demonstrated that IFNα activated STAT1, STAT2, and STAT3 in pancreatic β cells through TYK2, and that STAT2 was more critical than STAT1 in mediating the inflammatory and endoplasmic reticulum (ER) stress response (41). Another study likewise reported on IFNα induction of ER stress in pancreatic β cells, leading to the downregulation of insulin production and influence on T1D onset (42). A mouse model study revealed that inhibition of IFNα, but not IFNβ, in the pre-diabetes stage prevented the onset of T1D and blocked autoreactive T cells from entering and killing β cells in the pancreatic islets (43). Notably, patients with neutralizing autoantibodies to type I IFNs, specifically IFNαs, are less likely to develop T1D (44). These studies identify the negative impact of IFNα on the development of T1D.

Sjögren’s syndrome is an autoimmune disease with glandular lymphocyte infiltration leading to symptoms of dry mouth and eyes, where approximately 50% of patients have a type I IFN signature (45, 46). Given that this IFN signature is not present in all patients, one study analyzed the effects of IFN-α2b treatment of peripheral blood mononuclear cells (PBMCs) from patients with Sjögren’s compared with PBMCs from healthy donors, including type I IFN signature-positive and negative patients (45). Baseline effector protein phosphorylation levels differed predominantly in T cells in Sjögren’s patients compared with healthy individuals, with higher p-p38 and p-STAT1 (Y701, S727). Sjögren’s patients also exhibited increased IFNα-inducible JAK phosphorylation of STAT1 (Y701). Further, IFNα-2b treatment of PBMCs upregulated p-STAT1 (Y701) in B cells and downregulated p-STAT3 on S727 in T cells in type I IFN signature-positive patients.

SLE manifestations include organ damage and skin rash (47, 48). There is an IFNα signature in sera of SLE patients. A recent study using inducible IFNα transgenic mice found that upregulation of IFNα alone was capable of inducing an SLE phenotype (47). SLE pathogenesis is characterized by inflammasome overactivation; one study demonstrated that prolonged IFNα treatment increased inflammasome activity, which was eliminated with knockdown of IRF1 in SLE monocytes (49). IFNα treatment increased p-STAT1 and p-STAT2 at tyrosine residues, indicative of a classical JAK/STAT driven response. Another group that analyzed B cells from SLE patients, found increased baseline p-STAT3 (Y705), not p-STAT1, compared to B cells from healthy individuals (50). Additionally, these investigators found that IFNα treatment polarized naïve B cell differentiation towards a lupus-like phenotype, which was reversed by a STAT3 inhibitor and was absent in STAT3-deficient donor naïve B cells. In SLE monocytes, Gkirtzimanaki et al. identified IFNα induced mTOR activity, which promoted oxidative stress, revealing non-canonical IFNα signaling in SLE (51).

Cognizant of the persistent IFNα signature in SLE patients, a phase IIb clinical trial evaluated the effects of vaccination with IFNα kinoid, which produces anti-IFNα antibodies (52). Although the trial did not see a benefit in Based Composite Lupus Assessment (BICLA), the drug did provoke anti-IFN-α2b serum antibodies and decreased the IFN gene signature in 91% of patients. An anti-IFNAR1 monoclonal antibody, anifrolumab, has been evaluated in 11 clinical trials for SLE (9), Sjögren’s (1), and rheumatoid arthritis (1), with encouraging results (53). A recent phase III trial in SLE did not meet its primary endpoint of response, as per the SLE Responder Index; however, the same group conducted another phase III trial using the of British Isles Lupus Assessment Group (BILAG)-BICLA response as the primary endpoint and reported a statistically significant higher percentage of patients having a response as well as seeing a decrease in secondary endpoints, suggesting that a chronic IFNα response in SLE patients may contribute to disease pathogenesis (48).

Interestingly, while IFNα has been implicated in the pathogenesis of various autoimmune diseases, IFNβ has been used to successfully treat MS (54). Employing a mouse model of MS, studies with mice that lack the IFNβ gene revealed that in the absence of IFNβ the mice had a more severe disease with earlier onset and that the lack of IFNβ predisposed the mice to a pro-inflammatory Th17 immunophenotype (55, 56). Given the heterogeneity of the disease, and differing patient responses to IFNβ treatment, the identification of potential biomarkers of response to IFNβ therapy is receiving considerable attention. One study suggested predictors of response could be based on cell type-specific responses to type I IFN signaling, such as higher activation of STAT1, STAT3, and p38, leading to higher TRAIL expression in monocytes of IFN responders (57). Hurtado-Guerrero et al. analyzed monocytes from MS patients *ex vivo*, either left untreated (baseline) or after short-term IFNβ treatment (58). At baseline, there were no detectable differences in the levels of IFNAR1, IFNAR2, p-STAT1, and p-STAT2 among responders and non-responders, yet following IFNβ treatment, differences were observed. They found a pattern of decreased IFNAR1 and increased IFNAR2, p-STAT1, and p-STAT2 levels representing 68.4% of responder IFNβ-stimulated monocytes. Other groups have employed bioinformatics to uncover gene signatures that determine a response to IFNβ. One study used a feature selection computational method on a longitudinal microarray dataset of relapse-remitting MS (RRMS) patients treated with IFNβ-1b, and found a predictive seven gene signature (*CXCL9*, *IL2RA*, *CXCR3*, *AKT1*, *CSF2*, *IL2RB*, *GCA*) with 65.08% predictive accuracy (59). Using an alternative method of Elastic net modeling, Fukushima et al. analyzed time-course microarray datasets from PBMCs of MS patients and identified eleven (*ZBTB16*, *ZFP37*, *HPS5*, *HOPX*, *ARFGAP3*, *CALML5*, *VPS26A*, *SLC5A4*, *MBL2*, *DLGAP4*, *CACNA1C*) and eight (*SMA4*, *MIR7114*_*NSMF*, *LSM8*, *FLAD1*, *RRN3P1*, *RASL10A*, *IER3IP1*, *CDH2*) genes predictive of an IFNβ response, with 81% and 78% accuracy, respectively, for each dataset (60). A different study employed the GeneRank method to identify monotonically expressed genes (MEGs) that determine a good response (*AFTPH*, *ALOX5*, *ATG7*, *MYD88*, *LILRB1*, *PRKAB1*, *PSEN1*, *VAMP3*) and a bad response (*AGFG1*, *CHM*, *IGLL1*, *PELI1*, *PTEN*) for responders, and two bad response MEGs for non-responders (*NAP1L4*, *MMS19*) in IFNβ treated RRMS patients (61). As an alternative strategy to gene analysis, a logistic regression modeling method was used to examine metabolites from the sera of a cohort of MS patients to predict the production of anti-drug antibodies (ADA) to IFNβ treatment (62). Differences in 29 metabolites were shown to be indicative of ADA production, and the top ten most significant metabolites were lipid related. Another study using a systems immunology approach evaluated ADA production differences in three IFNβ treated cohorts and showed reduced baseline NOTCH2 expression and that a pro-inflammatory phenotype in monocytes was predictive of ADA development (63). Given the preceding, there is a need for further identification and characterization of biomarkers that are reproducibly predictive of an IFNβ response in RRMS patients.

The differences between IFNα and IFNβ in the generation of effects in autoimmune diseases requires additional analysis. Although both type I IFNs bind to and initiate signaling cascades through the dimeric IFNAR, they do differ in primary amino acid sequences and in binding affinity to the receptor which may account for varying impacts of the response on cells (54, 64). Binding affinity for IFNAR1 and IFNAR2 varies among IFNα subunits, with overall higher affinity for IFNAR2 over IFNAR1, and IFNβ has tighter binding to each receptor subunit than any of the IFNα subunits (64, 65). How the induced signaling can differ after the type I IFN ligand binds is not well understood but studies have shown differences further downstream in genes and transcription factor binding sites of IFNα versus IFNβ signaling, such as enrichment of IRF8 binding sites in IFNβ response (54). As previously mentioned, cell-type and disease state lead to variance in type I IFN signaling which is further complicated by the differences invoked by IFNα and IFNβ and requires further studies, especially to understand the protein signaling cascades after binding of type I IFNs to the IFNAR.

### Canonical and Non-Canonical IFN Signaling in Antiviral Responses

IFNs are critical effectors of an antiviral response in mammalian cells. Following viral infection, type I IFNs are produced by immune and non-immune cells, bind to and activate IFNAR, and signal through canonical and non-canonical pathways (66–68). An area of interest has been the involvement of the IFN system in the pathophysiology of Coronavirus Disease 19 (COVID-19).

Since the emergence of severe acute respiratory syndrome coronavirus (SARS-CoV) in 2003 and Middle East respiratory syndrome coronavirus (MERS-CoV) in 2012, therapeutic options for treatment have been limited (69). Type I IFNs are attractive therapeutic candidates because of their ability to clear virus through direct inhibition of viral replication of both DNA and RNA viruses and their effects on the activation of specific immune cell subsets to assist with viral clearance (70). Many viruses, including coronaviruses, evade an IFN antiviral response by inhibiting the production of type I and III IFNs (71–73). Scrutiny of the SARS-CoV genome identified the genes NSP1, NSP3, ORF3b, and ORF6 that are antagonists for type I IFNs, as well as the N protein (74). ORF6 not only inhibits the production of IFN but can also inhibit the expression of ISGs by inhibiting STAT1 nuclear translocation, through disruption of karyopherin-mediated transport. IRF3 is an important transcription factor necessary for IFNβ expression. The papain-like protease (PLpro), conserved in both SARS-CoV and SARS-CoV-2, inhibits the phosphorylation required for IRF3 homodimerization and nuclear translocation leading to its association with CBP/p300 and NF-κB for IFNβ expression (75–77). Comparing the gene sequences between SARS-CoV and SARS-CoV-2 for NSP3, ORF3b, and ORF6, revealed sequence differences that may contribute to the greater sensitivity of SARS-CoV-2 to type I IFNs (77). Konno et al. made the observation that ORF3b inhibits type I IFN induction more so in SARS-CoV-2 than in SARS-CoV, and a naturally arising SARS-CoV-2 variant exerts even greater antagonism of type I IFN induction by ORF3b (78). Accumulating data continue to provide further evidence of a blunted IFN response in COVID-19 cases (79–83).

Recently, data have emerged that indicate that SARS-CoV-2 is sensitive to the antiviral effects of both IFNα and IFNβ in cell culture assays, similar to the sensitivity of SARS-CoV *in vitro* (84–86). A pilot clinical study during the SARS outbreak of 2003 demonstrated that treatment with an IFNα resulted in reduced disease-associated impaired oxygen saturation and rapid resolution of lung abnormalities (87). The evidence of SARS-CoV-2 sensitivity to IFN treatment and accumulating clinical studies suggest that IFN treatment may have therapeutic benefits for COVID-19 (88). Early on in the pandemic, Zhou et al. provided evidence that treating COVID-19 patients with nebulized IFN-α2b with or without the antiviral drug, arbidol, accelerated viral clearance from the airways of infected patients and also reduced the circulating levels of the inflammatory cytokines, IL-6 and CRP (89). Following up from this exploratory study, there have been several clinical studies evaluating the therapeutic benefit of IFNα and IFNβ treatment for COVID-19 (see **Table 2**). *In vitro* studies suggested greater antiviral effectiveness of IFNβ over IFNα against SARS CoV (97). This prompted the WHO SOLIDARITY randomized controlled trial of a combination of lopinavir/ritonavir, ribavirin, and IFNβ-1b versus lopinavir/ritonavir in SARS-CoV-2 (90). The findings suggest that the triple combination treatment was more effective than lopinavir/ritonavir alone, reducing symptom severity and time to viral clearance. Given the emerging evidence that lopinavir/ritonavir treatment may be ineffective against SARS-CoV-2, the ongoing trial had been amended to compare the therapeutic effectiveness of IFNβ with remdesivir, a viral polymerase inhibitor that has demonstrated limited therapeutic efficacy in COVID-19 cases. A prospective observational study was conducted to assess the therapeutic efficacy of IFN-α2b in SARS-CoV-2 patients during the first month after the COVID-19 outbreak began in Cuba. Intramuscular administration of IFN-α2b improved both the rate of recovery and case fatalities (91). However, a retrospective cohort study demonstrated that there is great importance on the timing of administration of IFN-α2b with reduction of in-hospital mortality when administered the first five days of admission but increased mortality and delayed recovery was seen if given later (92). Additionally, inborn errors of type I IFNs and presence of autoantibodies against type I IFNs can be determinants of severity of disease and effectiveness of type I IFN treatment (95, 98). Roughly 10% of COVID-19 patients with severe pneumonia in a cohort of 987 patients had neutralizing autoantibodies against IFNα, IFNω, or both, where patients with no or mild symptoms had no detectable autoantibodies (95). These findings demonstrate that administration of IFNα may not be effective in patients with severe condition and autoantibodies, but since IFNβ autoantibodies were uncommon in the same patients, IFNβ may provide a more beneficial treatment. The same group analyzed a separate cohort of patients with life-threatening pneumonia and found 3.5% had inborn errors in type I IFN related genes, specifically in loci pertaining to TLR3- and IRF7-dependent type I IFN induction (98). This showed a commonality with influenza since similar type I IFN related gene defects have been demonstrated in life-threatening influenza pneumonitis (99).

**Table 2:** Clinical studies involving type I interferons in SARS-CoV-2.

Type I IFN (administration or collection)	Other drugs in combination****	Study type****	Outcomes if applicable****	Trial # (reference)****
IFNα-2b (nebulized)	Umifenovir	Uncontrolled, exploratory cohort study	IFN-α2b ( ± arbidol) reduced time to viral clearance and circulating inflammatory cytokine (IL-6, CRP) levels	(89)
IFNβ-1b (subcutaneous)	Lopinavir/Ritonavir Ribavirin	Randomized controlled phase 2 trial	Triple combination treatment more effective than lopinavir/ritonavir alone, reducing symptom severity and time to viral clearance	NCT04276688 (90)
IFNα-2b (intramuscular)	Lopinavir/Ritonavir Chloroquine	Multicenter prospective study	Higher proportion of patients discharged from hospital in IFN-treated vs. non-IFN treated group	RPCEC00000318—Cuban Registry (91)
IFNα-2b (nebulized)	Lopinavir/Ritonavir Umifenovir	Retrospective cohort study	Early IFN-α2b administration reduced in-hospital mortality but increased mortality and delayed recovery with late administration (>5 days post hospital admission)	(92)
IFNα	Lopinavir/Ritonavir Ribavirin	Retrospective, single-center study	Time to clearance positively correlated with length of hospital stay in patients treated with IFN-α+lopinavir/ritonavir (± ribavirin)	(93)
IFNβ-1b IFNβ-1a (subcutaneous)	Hydroxychloroquine Lopinavir/Ritonavir	Single center randomized controlled phase 2 clinical trial	Completed—no results posted	NCT04343768 (94)
IFNβ-1b (subcutaneous)	Hydroxychloroquine	Prospective open-label randomized controlled phase 2 trial	Completed – no results posted	NCT04350281
IFNα-2b (nebulized)	Ganovo Ritonavir	Open controlled phase 4 trial	Completed – no results posted	NCT04291729
IFNβ-1a (subcutaneous)	Remdesivir	Adaptive randomized double-blind multicenter placebo-controlled phase 3 trial	Recruiting, Adaptive COVID-19 Treatment Trial 3	NCT04492475
IFNα IFNκ (plasma and serum),		Observational study	Autoantibodies for IFNα, IFNκ, or both found in 101 of 987 patients with life-threatening pneumonia, none in 663 patients with no or mild symptoms, 4 of 1227 healthy patients	(95)
ISGs (bronchoalveolar lavage fluid)		Observational study	COVID-19 patients had higher expression of ISGs with a proinflammatory subset, compared to healthy and pneumonia patients	(96)

Similar to SARS-CoV, SARS-CoV-2 interacts with the angiotensin-converting enzyme 2 (ACE2) for cell entry, while MERS-CoV exploits the dipeptidyl peptidase 4 (DPP4) receptor for entry into human cells (100–102). Ziegler et al. demonstrated that nasal secretory cells (goblet cells), type II pneumocytes, and absorptive enterocytes of the ileum are positive for the two critical receptors for SARS-CoV-2 cell entry, ACE2 and the type II transmembrane serine protease, TMPRSS2 (103). Their observation that ACE2 expression is induced by type I IFNs in primary upper airway basal cells and lung tissue is hard to reconcile with IFNs inhibiting infection by SARS-CoV-2, yet recent emerging data suggesting a role for the renin-angiotensin pathway in protection from specific clinical features of COVID-19 would support a role for ACE2 in limiting COVID-19 severity. The inability of mice to uptake SARS-CoV-2 infection through the mouse ortholog of entry receptor ACE2 prompted Israelow et al. to create an adeno associated virus-mediated human ACE2 mouse model that can be utilized to analyze SARS-CoV-2 in mice, and they found increased type I IFN signaling ISGs in the lungs and limited control of SARS-CoV-2 replication by type I IFNs (104). The involvement of canonical versus non-canonical pathways in the induction of IFN-responses against SARS-CoV2 remains to be elucidated.

SARS-CoV-2 and influenza viruses are respiratory infections where disease severity results in lung hyper-inflammation and acute respiratory distress. Findings from clinical studies suggest that the early viral phase of both infections is associated with a blunted IFN response, yet progression to severe disease shows no such failed IFN response, specifically elevated levels of ISGs in PBMCs are observed (105–107). The implications are that the therapeutic benefits of IFN treatment are applicable in the early viral phases of COVID-19 and influenza, but that once the pulmonary phases of both infections progress to hyper-inflammation, IFN treatment is likely to be contra-indicated. Non-canonical effects in type I IFN signaling in influenza have been demonstrated as with p38 MAPK signaling, shown to be important in affecting type I IFN production and signaling in highly pathogenic avian influenza virus infected endothelial cells (108). Additionally, IFN-κ treatment inhibits influenza replication in lung cells, dependent on IFNAR, p38, CHD6, and Fos activation, but not STAT1 (109). Notably, IFNα induced STAT3 activation is crucial for inhibition of influenza viral replication and ISG transcription in mouse embryonic fibroblasts (110).

Although antiretroviral therapy (ART) for Human Immunodeficiency Virus (HIV) infection has transformed this infection from a fatal one to a chronic disease, viral reservoirs complicate efforts for HIV elimination, and a recent review paralleled HIV reservoir persistence to immuno-editing and immune evasion in cancer (111). The roles of type I IFNs in the pathophysiology of HIV infection are not fully understood, but IFNα has been implicated as an adverse factor in the persistence of HIV-1. When circulating levels of IFNα were measured for healthy donors, primary-infected, and chronically-infected patients, higher IFNα levels were associated with higher viral loads and higher expression of the ISG, *USP18*, which negatively regulates IFNα signaling by displacing JAK2 bound to IFNAR2 (112). Humanized mouse models have provided evidence that whereas type I IFNs suppress early HIV infection, type I IFN signaling induces T cell depletion and impaired functionality during persistent infection. When IFN signaling is blocked in HIV-infected mice or in monkeys receiving ART, this reduces the HIV reservoir, rescues anti-HIV T cells, and reduces HIV-induced inflammation (113–115). Notably, HIV-1 proteins, Vpu and Nef, inhibit ISG expression through canonical IFNα mediated JAK/STAT1 signaling, blocking any antiviral benefits from IFNα (116). Knockout of IFNAR1 in an HIV-induced brain injury mouse model provided memory benefits and neuronal injury protection while suppressing p38 activation, indicating involvement of type I IFN non-canonical signaling in HIV-1–related neurotoxicity (117). Indeed, there is accumulating evidence that sustained type I IFN signaling, surprisingly, can promote viral replication for a number of viruses, mediated by induction of certain ISGs and inhibition of IRFs (14). IFN induced 2′5′-oligoadenylate synthetase-like (OASL) limits RNA virus replication through enhancing RIG-I signaling yet inhibits cGAS and promotes viral replication for DNA viruses such as HSV.

Of late, there are emergent data that SLFN proteins, non-canonical effectors of type I IFN signaling, have a role as antivirals. IFN induced SLFN11 expression controls protein synthesis by regulating tRNA abundance, limiting West Nile virus, dengue virus, and Zika virus replication, all (+) ssRNA viruses, but having little effect on (–) ssRNA viruses (118). Interestingly, SLFN 11 control of HIV-1 infection is independent of type I IFN signaling (119). IFNβ induced SLFN14 exhibits antiviral activity in mouse macrophages, limiting infection with influenza virus or the DNA virus, varicella-zoster virus (120).

Besides the duration of type I IFN signaling influencing whether there is inhibition or enhancement of viral replication (105, 112), cell environmental factors also contribute to a type I IFN response. In a mouse model of vesicular stomatitis virus infection, high salt levels augment type I IFN signaling through the non-canonical p38 pathway (121). In neurons, viral infection may cause pain hypersensitivity; type I IFNs elicit pain sensitization in neurons, by promoting MAPK interacting kinase phosphorylation of eukaryotic initiation translation factor (122).

## Conclusions and Future Expectations

Though over sixty years have elapsed since the original discovery of IFNs, in recent years, there has been mounting evidence for the critical roles of type I IFNs as immune regulators in multiple biological systems. The mechanisms of induction of type I IFNs and their subsequent biological responses are complex, due in part to the large number of family members, both cell type-dependent and independent biological responses, and varying influences in different disease settings. As identified above, for acute and chronic virus infections, type I IFN signaling can have distinct and sometimes contrasting biological effects. In malignancies, type I IFNs induce antiproliferative and antineoplastic effects but may also upregulate PD-L1 expression, thereby limiting an anti-tumor immune response. In some autoimmune diseases, such as SLE, the persistent exposure of immune cells to endogenous IFNα appears associated with pathogenesis. On the other hand, IFNβ provides therapeutic benefits in MS. Regardless of whether type I IFN associated responses contribute to favorable or poor outcomes, it is clear that both canonical and non-canonical IFN signaling pathways are critical for type I IFN responses. In many cases, both canonical and non-canonical are activated in parallel, but it is possible that in certain cell-type and disease states a given pathway may play a predominant role. With the identification of the roles of non-canonical MAPK and mTOR pathways, the involvement of PKC and SLFN proteins, our understanding of how type I IFN signaling alters the transcriptome to produce proteins that affect changes in biological responses has increased dramatically. The discovery of new non-canonical pathways and effectors has substantially advanced the field, but other non-canonical pathways may have yet to be identified. Understanding how there is connectivity between the classical, canonical JAK/STAT signaling, and non-canonical pathways will provide the basis for further targeting of type I IFN signaling in different diseases.

## Author Contributions

All authors have contributed in the writing and editing of this review. All authors contributed to the article and approved the submitted version.

## Funding

The research of LP is supported by grants CA77816 and CA121192 by the NIH. REP is supported in part by NIH/NCI training grant T32 CA070085. 

## Conflict of Interest

The authors declare that the research was conducted in the absence of any commercial or financial relationships that could be construed as a potential conflict of interest.
